# Elevated serum CA72-4 levels predict poor prognosis in pancreatic adenocarcinoma after intensity-modulated radiation therapy

**DOI:** 10.18632/oncotarget.3562

**Published:** 2015-03-12

**Authors:** Peng Liu, Yuan Zhu, Luying Liu

**Affiliations:** ^1^ Department of Radiotherapy, Zhejiang Cancer Hospital, Hangzhou, Zhejiang, China

**Keywords:** Pancreatic adenocarcinoma, CA72-4, Prognosis, Radiotherapy

## Abstract

Carbohydrate antigen 72-4 (CA72-4) is a human tumor-associated glycoprotein, commonly used as a tumor marker for diagnosing and predicting outcome in gastric and ovarian cancers. However, the relationship between serum CA72-4 levels and prognosis of pancreatic adenocarcinoma has not been fully elucidated. A total of 113 consecutive locally advanced pancreatic adenocarcinoma patients who underwent intensity-modulated radiation therapy (IMRT) with or without chemotherapy were enrolled in this study. Serum CA72-4 levels were analyzed using immunoenzymometric assays. The association between serum CA72-4 levels and prognosis was evaluated. Serum CA72-4 levels was related with lymph node metastasis (P<0.001). The median overall survival time was 14.0 months for patients with serum CA72-4 normal levels and 10.0 months for the elevated levels (P<0.001). Multivariate analysis identified that Serum CA72-4 concentration was a significant prognostic factor (P<0.001). The hazard ratio (HR) of elevated serum CA72-4 levels compared with normal serum CA72-4 levels was 2.34 (95% confidence interval [CI]: 1.46-3.73), after adjusted for gender and age. Based on this finding, Serum CA72-4 is a potential marker to predict lymph node metastasis and prognosis in pancreatic adenocarcinoma.

## INTRODUCTION

Pancreatic adenocarcinoma is one of the most extremely malignant neoplasms in both developing and developed countries. The incidence has been increasing in recent years, recorded rate of 8 per 100 000. A great number of pancreatic adenocarcinoma patients experienced disease progression in a very short time. Radical surgery with adjuvant chemotherapy is thought to be the single modality that can provide opportunity of cure and long-time survival [1]. However, due to poor early diagnosis, only 13-20% of patients are at stage I at the time of diagnosis. For locally advanced pancreatic adenocarcinoma, combined or sequential chemotherapy and radiotherapy is the standard method [2, 3]. Chemoradiation therapy displays a modest survival beneﬁt compared with radiotherapy or chemotherapy alone [4, 5]. However, some patients with similar clinical stage have remarkably different survival prognosis. In this way, heterogeneity of protein expression proﬁles may play a very important role in the development of pancreatic adenocarcinoma [6]. Various molecular biomarkers are useful to identify the biological characteristics and predict the behavior of pancreatic adenocarcinoma. However, to date, most of these markers had not been proven to be sufficiently effective [7].

Carbohydrate antigen 19-9 (CA19-9) is considered to be the best prognostic serum marker for pancreatic cancer [8]. Increased baseline CA19-9 is related with low resectability, early metastasis and short survival time. However, the clinical utility of CA19-9 to identify the patients who are at high risk for disease progression is still limited [9]. Carbohydrate antigen 72-4 (CA72-4) is a human tumor-associated glycoprotein of high molecular weight, detected by monoclonal antibody CC49 and B72.3. CA72-4 is commonly used as a tumor marker for diagnosing and predicting outcome in gastric [10] and ovarian cancers [11]. CA72-4 is shown to provide independent prognostic information in pancreatic cancer postoperation. In Louhimo J et al's study [12], CA72-4 is superior to CA19-9 in predicting the outcome of pancreatic cancer. To our knowledge, no report has been published regarding the relationship between serum CA72-4 levels and clinicopathological features and prognosis of elocally advanced pancreatic adenocarcinoma treated with intensity-modulated radiation therapy (IMRT). Therefore, the objectives of this study were (1) to examine serum CA72-4 levels in 113 locally advanced pancreatic adenocarcinoma, (2) to assess the association between serum CA72-4 levels and clinicopathological features in locally advanced pancreatic adenocarcinoma, (3) to evaluate the prognostic impact of serum CA72-4 levels on overall survival in patients with locally advanced pancreatic adenocarcinoma.

## RESULTS

This survival study was carried out in 113 consecutive locally advanced pancreatic adenocarcinoma patients. The baseline characteristics of these patients are summarized in Table 1. A total of 71 men and 42 women were included with ages ranging from 34 to 78 years (median, 60 years). Primary tumor site was head in 57 patients, body and tail in 56 patients. There were 14 (12.4%) T_1_, 15 (13.3%) T_2_, 47 (41.6%) T_3_, and 37 (32.7%) T_4_. In terms of the new AJCC TNM staging system, 60 patients (53.1%) were categorized as N_0_ and 53 (46.9%) as N_1_. All the patients were included in the survival analysis. The overall follow-up durations ranged from 3 to 45 months (median, 12.0 months). 94 of 113 patients died of disease progression.

**Table 1:** Relationship between serum CA72-4 levels and clinicopathological variables (n=113)

		serum CA72-4 levels		P
Variables	Total (n)	CA 72-4≤6.9 U/mL (n=71)	CA 72-4>6.9 U/mL (n=42)	
Gender				
Male	71	41 (57.7%)	30 (42.3%)	0.146
Female	42	30 (71.4%)	12 (28.6%)	
Age				
<65	80	52 (65.0%)	28 (35.0%)	0.458
≥65	33	19 (57.6%)	14 (42.4%)	
Smoking status				
Never	58	44 (75.9%)	14 (24.1%)	0.003
Current/ever	55	27 (49.1%)	28 (50.9%)	
Tumor location				
Head	57	35 (61.4%)	22 (38.6%)	0.322
Body and tail	56	36 (64.3%)	20 (35.7%)	
T stage				
T_1-2_	29	16 (55.2%)	13 (44.8%)	0.322
T_3-4_	84	55 (65.5%)	29 (34.5%)	
N stage				
N_0_	60	47 (78.3%)	13 (21.7%)	<0.001
N_1_	53	24 (45.3%)	29 (54.7%)	
Treatment Response				
CR+PR	37	27 (73.0%)	10 (27.0%)	0.120
SD+PD	76	44 (57.6%)	32 (42.1%)	

**Table 2:** Univariable analysis for the effect of serum CA 72-4 levels on overall survival (n=113)

Variables	Univariable analysis		
	HR	95% CI	P
Gender (Male Vs Female)	0.84	0.55-1.29	0.424
Age (<65 Vs≥65)	0.93	0.59-1.45	0.740
Tumor location (Head Vs Body and tail)	0.78	0.52-1.18	0.242
T stage (T_1-2_ Vs T_3-4_)	0.97	0.61-1.54	0.897
N stage (N_0_ Vs N_1_)	0.99	0.66-1.48	0.942
Smoking status (Current, ever Vs Never)	1.98	1.29-3,03	0.002
Chemotherapy (Yes Vs No)	0.72	0.48-1.09	0.125
Treatment response (CR+PR Vs SD+PD)	4.81	2.87-8.05	<0.001
CA 72-4 levels (CA 72-4>6.9 U/mL Vs CA 72-4≤6.9 U/mL)	2.36	1.53-3.65	<0.001

**Table 3:** Multivariable analysis for serum CA 72-4 levels on overall survival (n=113)

Variables	Multivariable analysis		
	HR	95% CI	P
Smoking status (Current, ever Vs Never)	1.26	0.81-1.97	0.297
CA 72-4 levels (CA 72-4>6.9 U/mL Vs CA 72-4≤6.9 U/mL)	1.97	1.27-3.08	0.003
Treatment response	4.30	2.55-7.27	<0.001
After adjusted for gender and age			
Smoking status (Current, ever Vs Never)	1.25	0.80-1.95	0.332
CA 72-4 levels (CA 72-4>6.9 U/mL Vs CA 72-4≤6.9 U/mL)	2.34	1.46-3.73	<0.001
Treatment response	5.52	3.17-9.62	<0.001

Seven patients (6.2%) achieved a complete response (CR) as the best response post-treatment. The median overall survival time was 36 months. Thirty patients (26.5%, 14 in head of pancreas and 16 in body and tail of pancreas) had a partial response (PR) with median overall survival time 15.5 months. The clinical benefit rate, calculated as CR+PR, was 32.7%. 68 patients (60.2%) had stable disease (SD). The median overall survival time was 8 months. Progressive disease (PD) was observed in 8 patients (7.1%). The median overall survival time was only 7 months.

### Relationship between serum CA72-4 levels and clinicopathological parameters

The relationship between serum CA72-4 levels and clinicopathological parameters is presented in the Table1. The median of serum CA72-4 levels in all patients was 4.05 U/mL (range: 0.10–173.61 U/mL). Forty-two patients (37.2%) had serum CA72-4 levels more than 6.9 U/mL, defined as elevated group according to the manufacture's instruction. A cut-off point of 65 years was established to determine whether age affects serum CA72-4 levels and overall survival. There was no relationship between serum CA72-4 levels and patients' age (P>0.05). Serum CA72-4 levels were significantly higher in males than in females (mean, 11.9 U/mL Vs 10.4 U/mL, P<0.05). The Chi-square test also exhibited that there was near to be significant difference between male patients and female patients (P=0.146). Serum CA72-4 levels were also related with N stage (P<0.001). There was no significant correlation between serum CA72- levels and tumor location and T stage (P>0.05). Serum CA72-4 levels in smoking pancreatic adenocarcinoma patients were significantly higher than never smoking patients (mean: 13.15 U/mL Vs 2.46 U/mL, P=0.004). Serum CA72-4 levels did not relate with treatment response (P=0.120), even though circulating CA72-4 levels were slightly higher in patients with CR+PR than those with SD+PD (t-test, P=0.136).

### Serum CA72-4 levels and patients' prognosis with univariable and multivariable analysis

**Figure 1:**
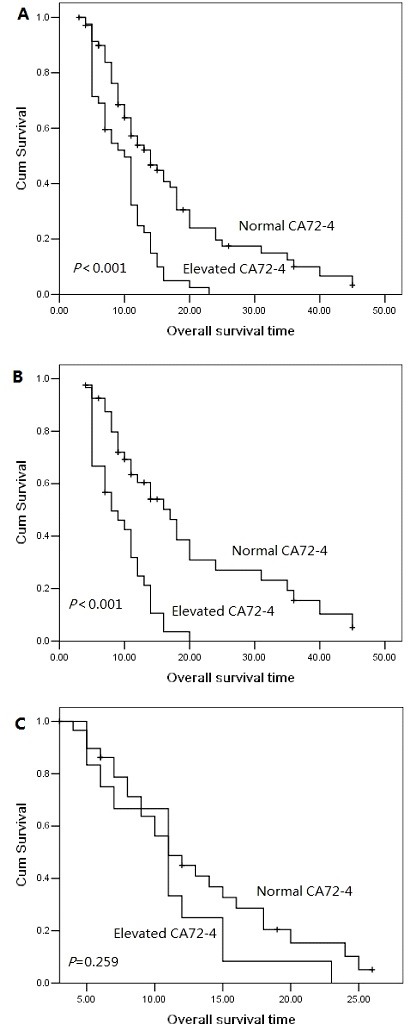
Overall survival curves with serum CA72-4 levels in the entire population (A), in female patients (B) and in male patients (C).

**Figure 2:**
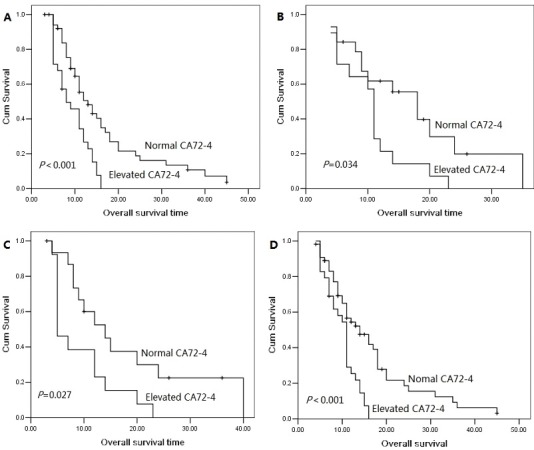
Overall survival curves with serum CA72-4 levels in age <65 patients (A), age≥65 patients (B), stage T1-2 patients (C) and stage T3-4 patient (D).

**Figure 3:**
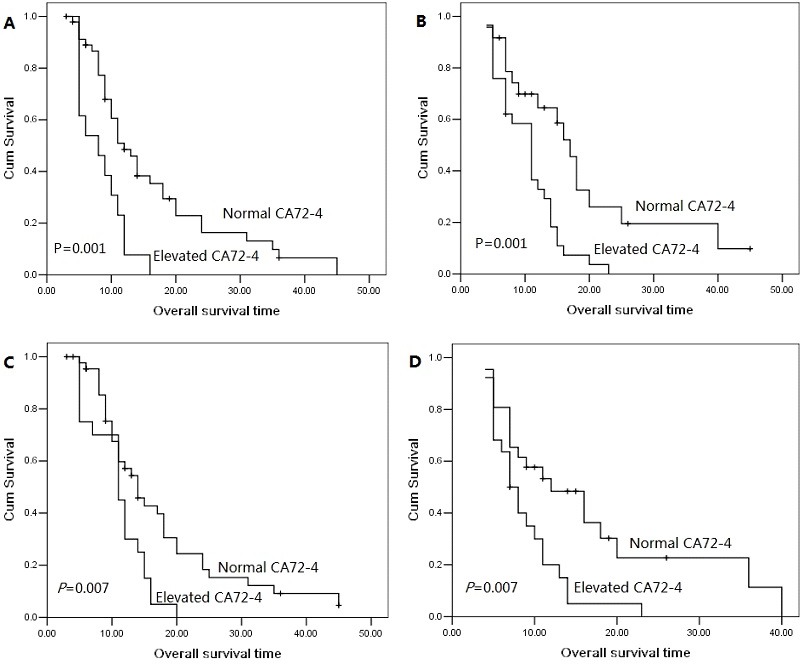
Overall survival curves with serum CA72-4 levels in stage N0 patients (A), in stage N1 patients (B), in patients with chemotherapy (C), in patients without chemotherapy (D).

The 1 year, 2 year and 3 year cumulative overall survival (OS) rates were 42.4%, 13.8% and 5.7%, respectively. The median overall survival time was 14.0 months for patients with serum CA72-4 normal levels and 10.0 months for elevated levels. The 2 year overall survival rate was 19.6% for patients with serum CA72-4 normal levels and 0% for elevated levels, indicating a significantly worse prognosis in serum CA72-4 elevated levels (P<0.001, Figure 1A). The median overall survival time was shorter in patients with serum CA72-4 elevated levels than in those with normal levels in male pancreatic adenocarcinoma patients (8 months Vs 17 months, P<0.001, Figure 1B). However, in the population of female patients, there was no significant difference of median OS between those with serum CA72-4 normal levels (median OS=11 months) and those with serum CA72-4 elevated levels (median OS=11 months) (P=0.259) (Figure 1C). Two-year survival probability was 0% for serum CA72-4 elevated levels patients versus 18.8% for serum CA72-4 normal levels patients with age <65 years (P=0.004, Figure 2A). Similarly, patients with serum CA72-4 elevated levels had shorter OS than those with serum CA72-4 normal levels in the patients with age ≥65 years (median OS: 11.0 months Vs 18.0 months, P=0.034, Figure 2B). There was also significant difference of OS between serum CA72-4 normal levels patients and serum CA72-4 elevated levels patients among stage T_1-2_ (Median OS: 14.0 months Vs 5.0months, P=0.027, Figure 2C) and stage T_3-4_ (Median OS: 14.0 months Vs 11.0 months, P<0.001, Figure 2D). Furthermore, we examined the overall survival differences of patients stratified for normal CA72-4 levels and elevated CA72-4 levels according to regional lymph node metastasis. We found that for patients without lymph node metastasis, a highly significantly inferior outcome was observed in patients with elevated serum CA72-4 levels, compared with normal serum CA72-4 levels (median OS: 8 months Vs 12 months, P=0.001, Figure 3A). Also, there was significant difference in OS between patients with elevated serum CA72-4 levels and normal serum CA72-4 levels (median OS: 11 months Vs 17 months, P=0.001, Figure 3B) in the patients with positive lymph node metastasis. Elevated serum CA72-4 levels were also significantly associated with shorter OS for patients with chemotherapy (P=0.007, Figure 3C) and patients without chemotherapy (*P*=0.007, Figure 3D).

We performed univariate analysis for serum CA72-4 levels and other 8 clinicopathological variables (including gender, age, tumor location, T stage, lymph node metastasis, smoking status, treatment response and chemotherapy) to find useful prognostic factors for pancreatic adenocarcinoma. Serum CA72-4 levels, treatment response and smoking status were three significant prognostic factors (serum CA72-4 levels: P<0.001; treatment response: P<0.001; smoking status: P=0.002). The hazard ratio (HR) of elevated serum CA72-4 levels compared with normal serum CA72-4 levels was 2.36 (95% confidence interval [CI]: 1.53-3.65), indicating that elevated serum CA72-4 levels increased 2.36-fold higher risk for cancer-related death. We then performed multivariate analysis for these factors whose presence significantly affected prognosis. Patients with elevated serum CA72-4 levels had a 1.97-fold increased risk of death compared to those with normal serum CA72-4 levels. The 95% CI was 1.27-3.08. When adjusted for patients' gender, age, the adjusted HR of elevated serum CA72-4 levels became 2.34 (95% CI: 1.46-3.73) in comparison with normal serum CA72-4 levels. These finding indicated that pretreatment serum CA72-4 is a potential marker to predict lymph node metastasis and prognosis in pancreatic adenocarcinoma.

## DISCUSSION

In this study, we find that elevated pre-treatment serum CA72-4 levels are associated with poorer prognosis in pancreatic adenocarcinoma patients receiving IMRT. Serum CA72-4 levels were significantly higher in smoking patients (P=0.003). Patients with elevated serum CA72-4 levels had 2.34 times the risk of death compared with those with normal serum CA72-4 levels adjusted for patients' gender, age. Furthermore, our study demonstrated that serum CA72-4 levels were significantly related with cancer cell lymph node metastasis, in agreement with previous reports in gastric cancer [13]. Commonly, lymphatic metastasis is an early event in pancreatic adenocarcinoma, which is a strong prognostic factor for pancreatic adenocarcinoma. These result indicated that CA72-4 may be involved in the process of pancreatic adenocarcinoma from local disease to systematic disease, which subsequently results in inferior outcome.

Jiang et al [14] found that serum CA72-4 was relatively higher in pancreatic cancer patients but lower in patients with pancreatic benign disease. Testing CA72-4 combined with CA19-9 may increase the sensitivity and specificity in the diagnosis of pancreatic cancer. Moreover, after radical pancreatic mass resection, serum CA72-4 levels would be decrease significantly. In a recent report [15], the specificity of diagnosis of the combined detection of CA19-9 and CA72-4 was increased to 92.8%. In Louhimo et al's study [12], preoperative serum CA72-4 concentration was an independent novel prognostic biomarker in operable pancreatic cancer. However, the study included both pancreatic adenocarcinoma and endocrine malignancy. In our study, serum CA72-4 levels were observed elevated in 37.2% patients (42/113), and most of them were male patients. Current/ever smoking status may be related with higher serum CA72-4 levels. The univariated and multivariated analysis revealed that serum CA72-4 levels were significantly related with patients' outcome receiving IMRT, with chemotherapy or without chemotherapy.

There are growing evidences to support the theory that CA72-4 plays important role in tumor cell survivorship and progression [16]. However, the exact reason between elevated serum CA72-4 levels and worse prognosis of pancreatic adenocarcinoma remains unknown. CA72-4 is rarely expressed in the most benign tumors and normal adult tissues. Overexpression of CA72-4 could be related with cancer incidents. Szekanecz et al [17] reported that CA72-4 may serve as cell adhesion molecules. Cell adhesion is considered to be a critical process in the migration and metastasis of pancreatic cancer. CA72-4, like other tumor antigens CA19-9 and CA125, may mediate tumor cell adhesion [18], involve in the metastasis and influence the prognosis of pancreatic adenocarcinoma patients. In our study, CA72-4 is associated with regional lymph node metastasis, revealing that CA72-4 may play a vital part in the tumor cell metastasis. Therefore, CA72-4 is involved in the regulation mechanism of tumor cell adhesion and its specific role in tumor cell metastasis is worthy of being further study.

The major limitation in this study is that the information on post-treatment local recurrence or metastasis was insufficient. One of the least convincing things in this study is lack the data of disease free survival, although overall survival is the standard indicator in the cancer prognosis study. Second, this retrospective study is relatively small sample size. A prospective study is required to determine the prognostic value of serum CA72-4 levels. Furthermore, this study used traditional method to determine the concentration of serum CA72-4, more sensitive and specific techniques, like real time RT-PCR or ultrasensitive electrochemical immunosensor [19] are needed.

In summary, the present study revealed that elevated serum CA72-4 levels are significantly related with metastasis and progression of pancreatic adenocarcinoma. Pre-treatment serum CA72-4 levels are new novel independent prognostic biomarker for overall survival in pancreatic adenocarcinma receiving IMRT with or without chemotherapy. This finding provides information of further probe into the biological relevance of CA72-4 in pancreatic adenocarcinoma.

## MATERIALS AND METHODS

A total of 122 consecutive locally advanced pancreatic adenocarcinoma patients who underwent IMRT with or without chemotherapy from January 2010 to December 2013 at Zhejiang Cancer Hospital. All patients were newly confirmed to have pancreatic adenocarcinoma and had not received treatment previously. Patients with other malignancies were excluded from this study. Finally, a total of 113 eligible patients enrolled in our study. Each case was reassigned for tumor, node and metastases (TNM stage) classification and clinical stage according to the American Joint Committee on Cancer (AJCC) staging system [20]. The following detail clinical information was retrospectively collected and analyzed for each case: gender, age at treatment, smoking status, tumor location, clinical TNM stage, treatment response and overall survival (OS) after IMRT. Our study was approved by the institutional review board of the hospital. All patients provided informed consent before IMRT. Overall survival was calculated as the time from radiotherapy to death or censoring.

Each patient provided 5-mL blood sample pretreatment. The drawn blood samples were stored at −20°C until analysis. Serum CA72-4 levels were analyzed using immunoenzymometric assays (Immuno 1, Bayer, Tarrytown, NY). The recommended cut-off value for diagnostic purpose was 6.9 U/mL for CA72-4.

### Treatment schedule

All patients were immobilized in a supine position, arms overhead, with thermoplastic cast. Intravenous and oral contrast-contrast CT-simulation was performed at 5mm intervals of abdomen using CT simulator (GE, Lightspeed, USA). Radiation plans for IMRT were generated using Pinnacle Version 8.0. All patients underwent external beam radiation therapy with 6 MV X-rays. The area of solid macroscopic tumors in pancreas, the surrounding tissue infiltrated and the regional lymph node metastasis were defined as the gross tumor volume (GTV). The GTV plus a margin of at least 5 mm, including any areas of microscopic spread and the regional lymph nodes (peripancreatic, celiac, superior mesenteric, portal hepatic, retroperitoneal), was defied as the clinical target volume (CTV). The plan target volume (PTV) was defined as the CTV plus 0.5-1.0cm to account for the daily setup variation and respiratory movement. A fractional daily dose of 1.8 Gy (5 days per week, over 5 weeks) at an isocenter was prescribed. The median delivered dose of IMRT was 50 Gy (ranged: 44.0-55.8Gy). The dose to the adjacent normal structures was constrained as follows: The liver dose was limited to V_5_<75%, V_20_<50%, V_30_<30% and a mean dose less than 28 Gy. The kidney dose was limited to V_12_<50% and V_22.5_<30%. Spinal cord maximum dose was held to 45 Gy. The PTV encompassed at least 95% isodose line. The dose volume histogram (DVH) was obtained for CTV, PTV, spinal cord, liver and kidney. The small bowel contour was confined to the small bowel loops within 3 cm of the PTV and was limited to maximal dose <54 Gy. Sixty-five patients received two cycles of gemcitabine or S-1 based concurrent chemotherapy. S-1 was administered at 50 mg/m^2^ twice daily from day 1 to day 14, and gemcitabine was administered by 30-min intravenous infusions of 1000 mg/m^2^ on day 1 and day 8 of a 21-d cycle.

The CR (complete response), PR (partial response), SD (stable disease) and PD (progressive disease) were assessed at an interval of at least 4 weeks to confirm the objective response. All patients received standardized follow-up, occurring at 3 months interval for two years, 6 months interval the third year, and yearly thereafter. Evaluations comprised a physical examination, complete blood count, liver and kidney function tests, abdominal ultrasound or CT, chest radiography and pelvic CT.

### Statistical analysis

A post-treatment follow-up of the patients was carried out in order to evaluate the impact of CA72-4 on the overall survival. Overall survival was defined as the time interval from the initial event (radiotherapy) to the death or censoring. Serum CA72-4 levels was analyzed as a continuous variable and a categorical variable after grouping by normal levels (≤6.9 U/mL) and elevated levels (>6.9 U/mL). The chi-square test was performed to evaluate the association between the clinicopathological variables and serum CA72-4 levels. Unpaired student's t-test was used to compare serum CA72-4 levels according to clinicopathological variables. Survival curves were estimated by the univariate Kaplan-Meier method. The log-rank test was applied to check the significant differences in the curves among groups. Furthermore, we used the Cox proportional hazards model with the backward selection method for multivariate analysis. All statistical calculations were performed with SPSS 13.0 for Windows (Chicago, IL). Two-sides P values of < 0.05 were considered statistical significance.
